# Transient receptor potential ankyrin 1 (TRPA1) is functionally expressed in primary human osteoarthritic chondrocytes

**DOI:** 10.1186/s13075-016-1080-4

**Published:** 2016-08-11

**Authors:** Elina Nummenmaa, Mari Hämäläinen, Lauri J. Moilanen, Erja-Leena Paukkeri, Riina M. Nieminen, Teemu Moilanen, Katriina Vuolteenaho, Eeva Moilanen

**Affiliations:** 1 The Immunopharmacology Research Group, University of Tampere School of Medicine and Tampere University Hospital, Tampere, Finland; 2 Coxa Hospital for Joint Replacement, Tampere, Finland

**Keywords:** Osteoarthritis, Chondrocyte, TRPA1, Inflammation, Matrix metalloproteinase

## Abstract

**Background:**

Transient receptor potential ankyrin 1 (TRPA1) is a membrane-associated cation channel, widely expressed in neuronal cells and involved in nociception and neurogenic inflammation. We showed recently that TRPA1 mediates cartilage degradation and joint pain in the MIA-model of osteoarthritis (OA) suggesting a hitherto unknown role for TRPA1 in OA. Therefore, we aimed to investigate whether TRPA1 is expressed and functional in human OA chondrocytes.

**Methods:**

Expression of TRPA1 in primary human OA chondrocytes was assessed by qRT-PCR and Western blot. The functionality of the TRPA1 channel was assessed by Ca^2+^-influx measurements. Production of MMP-1, MMP-3, MMP-13, IL-6, and PGE_2_ subsequent to TRPA1 activation was measured by immunoassay.

**Results:**

We show here for the first time that TRPA1 is expressed in primary human OA chondrocytes and its expression is increased following stimulation with inflammatory factors IL-1β, IL-17, LPS, and resistin. Further, the TRPA1 channel was found to be functional, as stimulation with the TRPA1 agonist AITC caused an increase in Ca^2+^ influx, which was attenuated by the TRPA1 antagonist HC-030031. Genetic depletion and pharmacological inhibition of TRPA1 downregulated the production of MMP-1, MMP-3, MMP-13, IL-6, and PGE_2_ in osteoarthritic chondrocytes and murine cartilage, respectively.

**Conclusions:**

The TRPA1 cation channel was found to be functionally expressed in primary human OA chondrocytes, which is an original finding. The presence and inflammatory and catabolic effects of TRPA1 in human OA chondrocytes propose a highly intriguing role for TRPA1 as a pathogenic factor and drug target in OA.

**Electronic supplementary material:**

The online version of this article (doi:10.1186/s13075-016-1080-4) contains supplementary material, which is available to authorized users.

## Background

Transient receptor potential ankyrin 1 (TRPA1) is a membrane-associated cation channel which mediates pain and hyperalgesia [1, 2] and functions as a chemosensor of noxious compounds [3–5]. TRPA1 was first discovered in 1999 [6] and has since then been found to be widely expressed in afferent sensory neurons, especially in Aδ and C fibers of nociceptors [7, 8]. In addition to pain, TRPA1 also has a role in mediating neurogenic inflammation [9, 10]. More recently, TRPA1 has been found to be expressed also in some nonneuronal cells such as keratinocytes [11] and synoviocytes [12] but the functional roles of nonneuronal expression remain to be studied.

TRPA1 is activated by numerous exogenous pungent compounds such as allyl isothiocyanate (AITC) from mustard oil [5], acrolein from exhaust fumes and tobacco smoke [9], and allicin from garlic [3]. Interestingly, TRPA1 is also activated and sensitized by agents formed endogenously in inflammatory reactions, such as nitric oxide [13], hydrogen peroxide [14] and nitro-oleic acid [15]. The activation of TRPA1 causes an influx of cation ions, particularly Ca^2+^, into the activated cells [16] and this elevation of intracellular Ca^2+^ has been shown to trigger an action potential in neuronal cells [16, 17]. Interestingly, among the many regulatory effects of the alterations of intracellular Ca^2+^ concentration, its increase has also been shown to affect the gene expression of inflammatory mediators [18–20].

Recent evidence suggests TRPA1 to have a role in inflammation through exogenous activation by TRPA1 agonists and also through endogenous mechanisms. TRPA1 has been shown to mediate carrageenan-induced inflammatory edema [21], tumor necrosis factor (TNF)-triggered hyperalgesia [22], airway hyperreactivity and inflammation [23, 24], and to relate to acute gouty arthritis [25, 26]. Very recently we found that TRPA1 has a role in mediating acute inflammation, cartilage destruction, and joint pain in monosodium iodoacetate (MIA)-induced inflammation and osteoarthritis in the mouse [27].

Osteoarthritis (OA) is the most common cause of musculoskeletal disability and pain worldwide and its prevalence is constantly increasing as the population ages. OA is a degenerative disease of the joints, which is characterized by inflammation and hypoxia within the joint, leading to cartilage degradation, joint deformity, disability, and pain [28, 29]. OA-related cartilage degradation is caused by a growing imbalance between the production of catabolic, anabolic, and inflammatory mediators within the joint driven by the increased expression of matrix-degrading metalloproteinases and proinflammatory mediators such as interleukin (IL)-6 and prostaglandin E_2_ (PGE_2_) [28].

TRPA1 has not previously been investigated in chondrocytes. However, factors involved in hypoxia and inflammation, such as hydrogen peroxide (H_2_O_2_), nitric oxide (NO), and IL-6 have been shown to upregulate the expression or activation of TRPA1 in some other cells [12–14]. Furthermore, the activation of TRPA1 has been reported to enhance the production of inflammatory factors [12, 21, 26, 30]. Since there is a hypoxic and inflammatory state in OA joints [28, 31], and TRPA1 has been shown to be involved in the mediation of acute inflammation and cartilage degradation in MIA-induced osteoarthritis [27], we hypothesized that TRPA1 is expressed in the chondrocytes in osteoarthritic joints, where its activation could play a vital part in the inflammation and pathogenesis of OA. In the present study, we tested that hypothesis by measuring the expression and function of TRPA1 in primary human OA chondrocytes.

## Methods

### Cell culture

Primary chondrocyte cultures were carried out as previously described [32]. Leftover pieces of OA cartilage from knee joint replacement surgery were used under full patient consent. The patients in this study fulfilled the American College of Rheumatology classification criteria for OA [33] and the study was approved by the Ethics Committee of Tampere University Hospital, Tampere, Finland (reference number R09116), and carried out in accordance with the Declaration of Helsinki. The procedures to isolate and culture the primary chondrocytes are described in the supplementary data (Additional file 1). During experiments the cells were treated with IL-1β (R&D Systems Europe Ltd, Abingdon, UK), IL-17 (R&D Systems Europe Ltd.), lipopolysaccharide (LPS) (Millipore Sigma, St. Louis, MO, USA), resistin (BioVision Inc., Milpitas, CA, USA), the TRPA1 antagonist HC-030031 (Millipore Sigma) or with combinations of these compounds as indicated.

Immortalized human T/C28a2 chondrocytes [34] were cultured as described in the supplementary data (Additional file 1). During the experiments T/C28a2 chondrocytes were treated with IL-1β (R&D Systems Europe Ltd), IL-17 (R&D Systems Europe Ltd.), LPS (Millipore Sigma), HC-030031 (Millipore Sigma) or with combinations of these compounds as indicated.

HEK 293 human embryonic kidney cells (American Type Culture Collection, Manassas, VA, USA) were cultured as described in the supplementary data (Additional file 1). The cells were transfected using 0.42 mg/cm^2^ of human TRPA1 plasmid DNA (pCMV6-XL4 by Origene, Rockville, MD, USA) with lipofectamine 2000 (Invitrogen, Life Technologies, Carlsbad, CA, USA) according to the manufacturer’s instructions.

### Animals

Wild-type (WT) and TRPA1 knockout (KO) male B6;129P-Trpa1(tm1Kykw)/J mice (Charles River Laboratories, Sulzfeld, Germany) aged 19–22 days were used in mouse cartilage culture experiments. Mice were housed under standard conditions (12–12 h light–dark cycle, 22 ± 1 °C) with food and water provided ad libitum. Animal experiments were carried out in accordance with the legislation for the protection of animals used for scientific purposes (Directive 2010/63/EU) and the experiments were approved by The National Animal Experiment Board (reference number UTA 845/712-86). Animals were sacrificed by carbon monoxide followed by cranial dislocation.

### Mouse cartilage culture

After mice were euthanized, full-thickness articular cartilage from femoral heads were removed and cultured as described in the supplementary data (Additional file 1). The cartilage pieces were exposed to IL-1β (R&D Systems Europe Ltd.) or its vehicle for 42 h and thereafter culture media were collected and matrix metalloproteinase (MMP)-3, IL-6, and PGE_2_ concentrations were measured by immunoassay.

### Western blot measurements

After the cell culture experiments, total protein was extracted, and TRPA1 was immunoprecipitated and analyzed with Western blot as described in the supplementary data (Additional file 1). TRPA1 antibody NB110-40763 (Novus Biologicals, LCC, Littleton, CO, USA) was used as the primary antibody and goat anti-rabbit HRP-conjugate (sc-2004, Santa Cruz Biotechnology, Inc., Dallas, TX, USA) as the secondary antibody in the Western blot analysis.

### Immunoassay

Concentrations of IL-6, MMP-1, MMP-3, MMP-13 and PGE_2_ in medium samples were determined by enzyme-linked immunosorbent assay (ELISA) with commercial reagents (PGE_2_: Cayman Chemical Co., Ann Arbor, MI, USA; human IL-6: eBioscience Inc. San Diego, CA, USA; MMP-1, MMP-3, MMP-13 and mouse IL-6: R&D Systems Europe Ltd).

### RNA extraction and quantitative RT-PCR

At the indicated time points, total RNA was extracted and analyzed by quantitative reverse transcription polymerase chain reaction (qRT-PCR) for the expression of TRPA1 mRNA as described in the supplementary data (Additional file 1).

### Ca^2+^-influx measurements

TRPA1-mediated Ca^2+^ influx was measured in HEK293 cells [35] transfected with human TRPA1 plasmid, in human T/C28a2 chondrocytes, and in primary human OA chondrocytes as described previously [36]. Briefly, the cells were loaded with 4 μM fluo-3-acetoxymethyl ester (Fluo-3-AM, Millipore Sigma) and 0.08 % Pluronic F-127® (Millipore Sigma in Hanks’ balanced salt solution (HBSS, Lonza, Verviers, Belgium) containing 1 mg/ml of bovine serum albumin, 2.5 mM probenecid and 25 mM HEPES pH 7.2 (all from Millipore Sigma) for 30 min at room temperature. The intracellular-free Ca^2+^ levels were assessed by Victor3 1420 multilabel counter (Perkin Elmer, Waltham, MA, USA) at excitation/emission wavelengths of 485/535 nm. In the experiments, the cells were first preincubated with the TRPA1 antagonist HC-030031 (100 μM, Millipore Sigma) or the vehicle for 30 min at +37 °C. Thereafter, the TRPA1 agonist allyl isothiocyanate (AITC, 50 μM, Millipore Sigma) was added and the measurements were continued for 30 s after which a robust Ca^2+^ influx was induced by application of the control ionophore compound ionomycin (1 μM, Millipore Sigma).

### Statistical analysis

Data were analyzed using Graph-Pad InStat version 3.00 software (GraphPad Software, San Diego, CA, USA). The results are presented as mean + standard error of the mean (SEM) unless otherwise indicated. Unpaired *t* test, paired *t* test, one-way analysis of variance (ANOVA) or repeated-measures ANOVA, followed by Dunnett’s test were used in the statistical analysis. Differences were considered significant at *p* < 0.05, *p* < 0.01, and *p* < 0.001.

## Results

### TRPA1 is expressed in primary human OA chondrocytes and in immortalized human T/C28a2 chondrocyte cell line

**Fig. 1:**
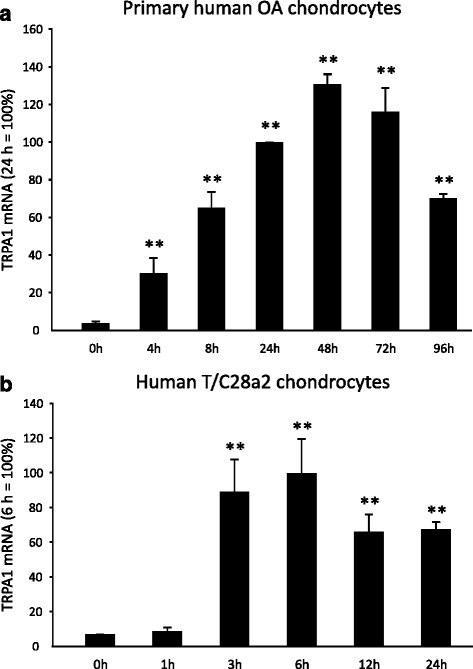
Primary human OA chondrocytes (**a**) and human T/C28a2 chondrocyte cell line (**b**) express TRPA1 mRNA and its expression is enhanced by IL-1 in a time-dependent manner. Cultures of primary human OA chondrocytes (**a**) and human T/C28a2 chondrocytes (**b**) were stimulated with IL-1β (100 pg/ml) for 0–96 h and 0–24 h, respectively, and thereafter total RNA was extracted. TRPA1 mRNA levels were measured by qRT-PCR, and the results were normalized against GAPDH mRNA. The mRNA levels are expressed as arbitrary units with the levels measured at 24 h (**a**; primary OA chondrocytes) or 6 h (**b**; T/C28a2 chondrocytes) set as 100 %; and the values at the other time points are related to those values. Primary chondrocyte samples were obtained from three to five different donors and the experiments were carried out in duplicate. Human T/C28a2 chondrocyte experiments were carried out in quadruplicate. Results are expressed as mean + SEM. One-way ANOVA followed by Dunnett’s post-test was used in the statistical analysis; ^**^indicates *p* < 0.01 compared to the control (0 h) sample. *OA* osteoarthritis, *TRPA1* transient receptor potential ankyrin 1

**Fig. 2:**
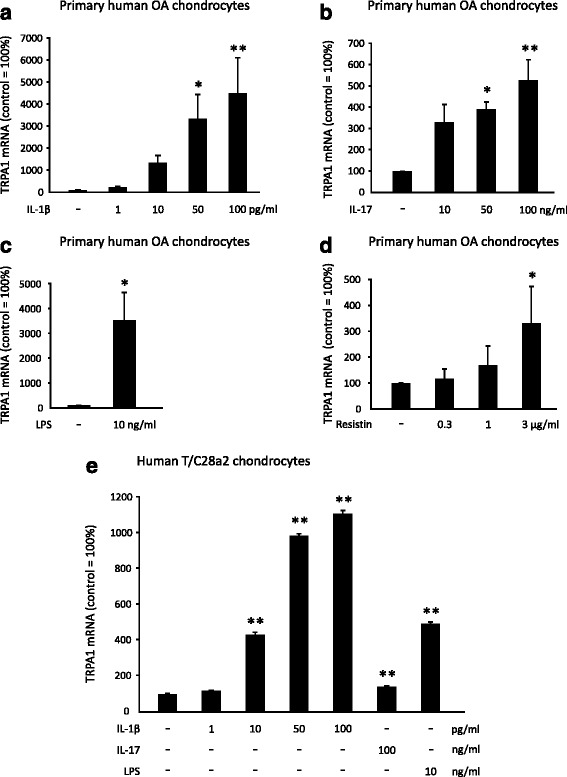
TRPA1 mRNA expression is increased following stimulation with inflammatory factors IL-1β, IL-17, LPS, and resistin in primary human OA chondrocytes (**a**-**d**) and in human T/C28a2 chondrocyte cell line (**e**). Isolated primary human OA chondrocytes were stimulated with IL-1β (1–100 pg/ml) (**a**), IL-17 (10–100 ng/ml) (**b**), LPS (10 ng/ml) (**c**). and resistin (0.3–3 μg/ml) (**d**); and human T/C28a2 chondrocytes with IL-1β (1–100 pg/ml), IL-17 (100 ng/ml) and LPS (10 ng/ml) (**e**) for 24 h; and thereafter total RNA was extracted. TRPA1 mRNA levels were measured by qRT-PCR, and the results were normalized against GAPDH mRNA levels. The results are expressed as a percentage in comparison to untreated control samples, which was set as 100 %. Primary chondrocyte samples were obtained from four different donors and the experiments were performed in duplicate. Human T/C28a2 chondrocyte experiments were carried out in quadruplicate. Results are expressed as mean + SEM. Repeated measures ANOVA (**a**, **b**, **d**) and one-way ANOVA (**e**) followed by Dunnett’s post-test or paired *t* test (**c**) was used in the statistical analysis; ^*^ *p* < 0.05 and ^**^ *p* < 0.01, compared to the untreated control samples. *IL* interleukin, *LPS* lipopolysaccharide, *OA* osteoarthritis, *TRPA1* transient receptor potential ankyrin 1

Primary human OA chondrocytes and immortalized human T/C28a2 chondrocyte cell line expressed TRPA1. The expression was measured by quantitative RT-PCR on isolated total mRNA using a specific TaqMan assay. The proinflammatory cytokine IL-1β was found to increase TRPA1 expression in a time-dependent manner: in primary chondrocytes the expression of TRPA1 increased up to 48 hours and declined thereafter (Fig. 1a), whereas in the human T/C28a2 chondrocytes the expression maximum was at 6 hours (Fig. 1b). In addition, TRPA1 expression was also enhanced by inflammatory factors IL-17, LPS, and resistin (Fig. 2).

**Fig. 3:**
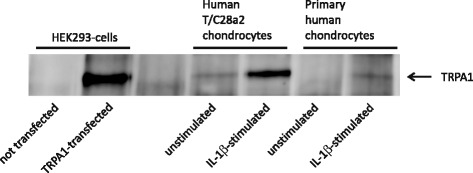
TRPA1 protein is expressed in primary human OA chondrocytes and human T/C28a2 chondrocyte cell line. Chondrocyte cultures were stimulated with IL-1β (100 pg/ml) for 24 h. Extracted proteins were immunoprecipitated and TRPA1 was detected with Western blot analysis. HEK293 cells transiently transfected with human TRPA1 plasmid were used as a positive control. Representative blot of three independent experiments with similar results. *IL* interleukin, *TRPA1* transient receptor potential ankyrin 1

To verify the translation of TRPA1 mRNA into protein, we extracted total protein from primary human OA chondrocytes and human T/C28a2 chondrocytes and performed Western blot analysis. HEK293 cells transiently transfected with TRPA1 plasmid were used as positive control and the protein was detected with a specific human TRPA1 antibody. Remarkably, both cell types were found to express TRPA1 protein as seen in Fig. 3.

### Human chondrocytes express a functional TRPA1 channel

**Fig. 4:**
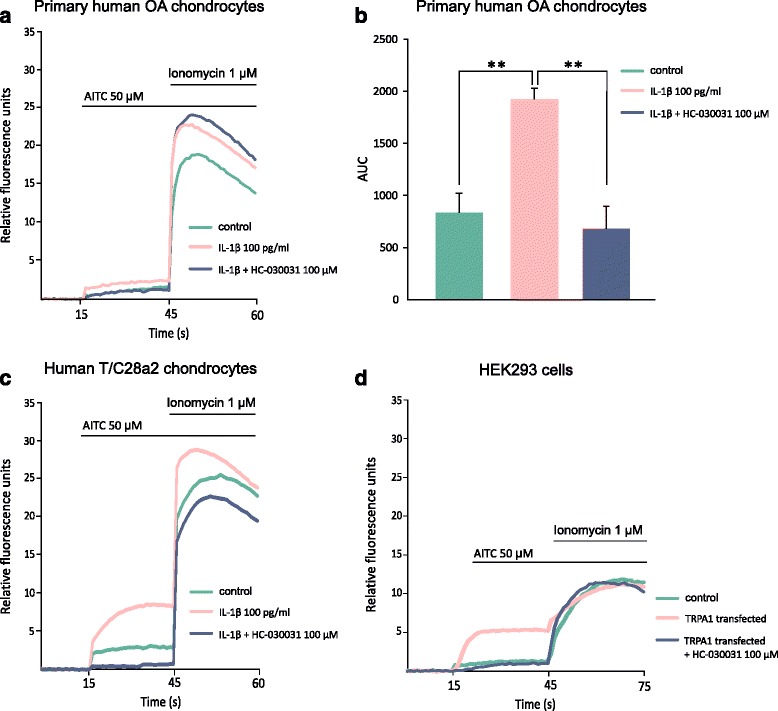
The TRPA1 ion channel is functional in primary human OA chondrocytes (**a**, **b**) and human T/C28a2 chondrocyte cell line (**c**) as shown by TRPA1-mediated Ca^2+^ influx. Primary human chondrocytes (**a**, **b**) and human T/C28a2 chondrocytes (**c**) were cultured with or without (control) IL-1β (100 pg/ml) for 24 h. HEK293 cells transfected with plasmids encoding human TRPA1 were used as positive control cells (**d**). The cells were loaded with Fluo-3-AM and the TRPA1-mediated Ca^2+^ influx was measured by Victor3 multilabel counter at excitation/emission wavelengths of 485/535 nm at 1/s frequency. The cells were first preincubated with the TRPA1 antagonist HC-030031 (100 μM) or the vehicle for 30 min at +37 °C. In the measurements, basal fluorescence was first recorded for 15 s and thereafter the selective TRPA1 agonist allyl isothiocyanate (AITC; 50 μM) was added and the measurement was continued for 30 s after which the control ionophore compound ionomycin (1 μM) was introduced to the cells. IL-1β stimulation resulted in an elevation in AITC-induced Ca^2+^ influx compared to unstimulated control cells, and it was attenuated by the selective TRPA1 antagonist HC-030031. The results were normalized against the background and expressed as mean of eight simultaneous measurements. Curves in *A*, *C* and *D* express results from one representative experiment. In (**b**) area under the curve (AUC) from 15 to 45 s was calculated from measurements of primary chondrocyte from four donors (each with eight repeats). Results are expressed as mean + SEM. Repeated measures ANOVA followed by Dunnett’s post-test was used in the statistical analysis; ^**^ *p* < 0.01 compared to the IL-1β-treated samples. *IL* interleukin, *OA* osteoarthritis, *TRPA1* transient receptor potential ankyrin 1

To confirm that TRPA1 mRNA and the subsequent protein expressed by human chondrocytes produces a functional channel, Ca^2+^-influx measurements were carried out. Primary human chondrocytes and T/C28a2 chondrocytes were cultured with IL-1β, which was found to stimulate TRPA1 expression, or with its vehicle for 24 h, and thereafter TRPA1 was activated with the TRPA1 agonist AITC. IL-1β stimulation resulted in an increased responsiveness to AITC as seen as an enhanced Ca^2+^ influx, and the selective TRPA1 antagonist HC-030031 was shown to prevent this effect (Fig. 4).

### MMP, IL-6 and PGE_2_ production is downregulated by genetic depletion and pharmacological inhibition of TRPA1

**Fig. 5:**
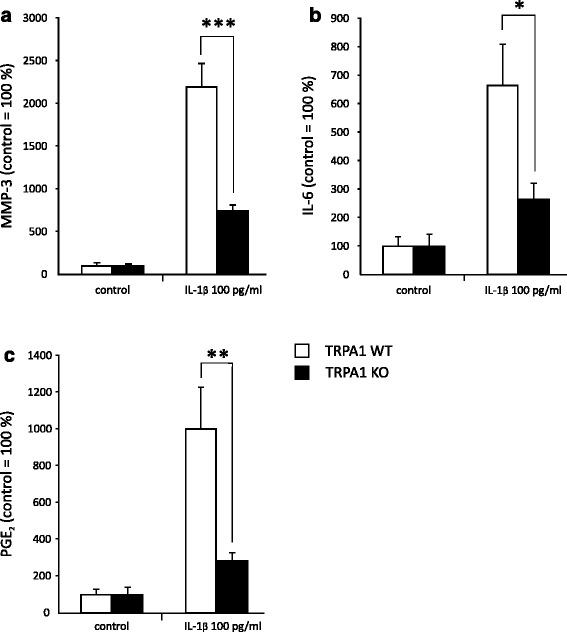
IL-1β-induced production of MMP-3 (**a**), IL-6 (**b**), and PGE_2_ (**c**) in the cartilage is attenuated by genetic depletion of TRPA1. Cartilage samples were obtained from TRPA1-deficient (knockout, KO) and corresponding wild-type (WT) mice. The samples were cultured with and without IL-1β (100 pg/ml) for 42 h and thereafter the culture medium was collected and analyzed for concentrations of MMP-3, IL-6, and PGE_2_ by immunoassay. The results are expressed as mean + SEM, n = 6–9. Unpaired *t* test was used in the statistical analysis; ^*^ *p* < 0.05, ^**^ *p* < 0.01, and ^***^ *p* < 0.001 compared to the WT mice. *IL* interleukin, *MMP* matrix metalloproteinase, *PGE*_*2*_ prostaglandin E_2_, *TRPA1* transient receptor potential ankyrin 1

**Fig. 6:**
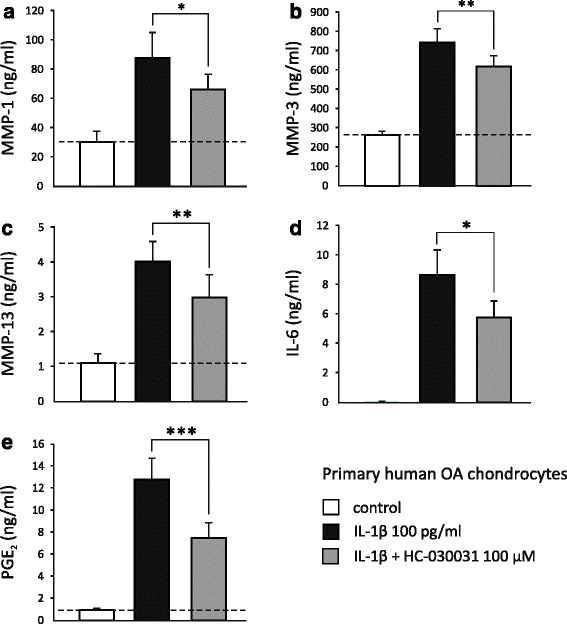
IL-1β-enhanced expression of MMP-1 (**a**), MMP-3 (**b**), MMP-13 (**c**), IL-6 (**d**), and PGE_2_ (**e**) in primary human OA chondrocytes is attenuated by pharmacological inhibition of TRPA1. Primary human OA chondrocytes were stimulated with IL-1β (100 pg/ml) in the presence and absence of the selective TRPA1 antagonist HC-030031 (100 μM) for 24 h. MMP-1, MMP-3, MMP-13, IL-6, and PGE_2_ concentrations in the culture media were measured by immunoassay and the results are expressed as mean + SEM. Samples were obtained from eight patients and the experiments were carried out in duplicate. Paired *t* test was used in the statistical analysis; ^*^ *p* < 0.05, ^**^ *p* < 0.01, and ^***^ *p* < 0.001 compared to the IL-1β-treated samples. *IL* interleukin, *MMP* matrix metalloproteinase, *OA* osteoarthritis, *PGE*_*2*_ prostaglandin E_2_, *TRPA1* transient receptor potential ankyrin 1

After finding that functional TRPA1 was indeed expressed in chondrocytes, we aimed to further examine the possible arthritogenic role of the TRPA1 channel. We investigated the effect of genetic depletion of TRPA1 on the production of OA-related factors MMP-3, IL-6, and PGE_2_ by using articular cartilage samples from TRPA1-deficient (knockout, KO) and corresponding wild-type (WT) mice. IL-1β treatment increased MMP-3, IL-6, and PGE_2_ production in cartilage as expected. Remarkably, this response was significantly attenuated in the cartilage from the TRPA1 KO mice as compared to the corresponding WT mice (Fig. 5). Further, we treated primary human chondrocytes with IL-1β alone and together with the selective TRPA1 antagonist HC-030031 for 24 h. Interestingly, the selective TRPA1 antagonist HC-030031 downregulated IL-1β-enhanced MMP-1, MMP-3, MMP-13, IL-6, and PGE_2_ production by 25–45 % (Fig. 6), suggesting that TRPA1 plays a role in the upregulation of these catabolic and inflammatory factors in OA cartilage.

## Discussion

The findings of the present study suggest a hitherto unknown role for TRPA1 in the pathogenesis of OA. We have shown for the first time the expression of the TRPA1 channel in primary human OA chondrocytes and in the human T/C28a2 chondrocyte cell line. We showed the expression of TRPA1 mRNA and protein by qRT-PCR and Western blot, respectively. We were also able to show that the expressed TRPA1 was functional, as evidenced by Ca^2+^-influx measurements. Further, we found TRPA1 to have a role in mediating the production of OA-related factors MMP-1, MMP-3, MMP-13, IL-6, and PGE_2_ as evidenced by pharmacological inhibition and genetic depletion of TRPA1.

TRPA1 was first discovered in 1999 in fetal lung fibroblasts [6]. Since then it has been mainly studied in different afferent sensory neurons such as Aδ and C fibers of nociceptors [7, 8]. More recently, however, TRPA1 has also been found to be expressed in some nonneuronal cells such as keratinocytes [11, 37, 38], synoviocytes [12, 39] and airway epithelial and smooth muscle cells [30]. It is noteworthy, that not all of these studies have shown functionality of the TRPA1 ion channel and some have only reported the expression of TRPA1 at the mRNA level. In the present study, we have comprehensively shown the expression and activation of TRPA1 in human chondrocytes, to support the criteria set by Fernandes et al. [40]. We were able to show for the first time the expression of both TRPA1 mRNA and protein and the functionality of the TRPA1 channel in primary human OA chondrocytes and in human T/C28a2 chondrocyte cell line. This finding is particularly interesting as in OA joints there is a hypoxic [31] and inflammatory [28, 41] state and related factors, H_2_O_2_, NO, and IL-6, have previously been shown to upregulate the expression and activation of TRPA1 [12–14]. According to Hatano et al. [12] the human *TRPA1* promoter has at least six putative nuclear factor kappa B (NF-kB) binding sites and ten core hypoxia response elements (HREs), which are binding sites for hypoxia-inducible factor (HIF) transcription factors. HIFs are known to mediate adaptive responses to hypoxia as well as to be activated by inflammation [42, 43] and the binding of HIFs to consensus HREs on their target genes regulates gene transcription.

After discovering TRPA1 expression in chondrocytes, we aimed to investigate whether inflammatory factors/mechanisms related to the pathogenesis of OA [28, 29] regulate expression of TRPA1, which would indicate a role for TRPA1 as a mediator in OA. IL-1β is considered as a major player in OA associated with cartilage destruction. IL-1β is elevated in OA joints and it suppresses type II collagen and aggrecan expression, stimulates the release of MMP-1, MMP-3, and MMP-13, and induces the production of IL-6 and some other cytokines as well as PGE_2_ [28]. In part IL-17 feeds forward these mechanisms as it further induces IL-1β, TNF, and IL-6 production, upregulates NO and MMPs and downregulates proteoglycan levels related to the pathogenesis of OA [28]. Based on our results, IL-1β and IL-17 both also induce TPRA1 expression and intriguingly, some of the IL-1β-induced inflammatory and catabolic effects are partly mediated by TRPA1. In OA the innate immune system and in particular toll-like receptors (TLRs) activated by cartilage matrix degradation products, also play a significant part in disease progression. Chondrocytes express TLRs, which trigger major inflammatory pathways and are activated by bacterial lipopolysaccharide (LPS) and damage-associated molecular patterns [29], and also the adipocytokine resistin known to be expressed in OA joints [44] has been shown to transduce its effects through toll-like receptor 4 [45]. In the present study, we found that both LPS and resistin increased expression of TRPA1 in human chondrocytes, suggesting a TLR-mediated mechanism to enhance TRPA1 expression in OA cartilage. In support of the present results, Hatano et al. showed that TRPA1 gene expression was enhanced in synoviocytes by inflammatory factors TNF-α and IL-1 [12], and the present study together with that of Hatano et al. [12] suggests a previously unrecognized mechanism that links TRPA1 as an inducible factor to joint inflammation.

Activation of TRPA1 results in a substantial influx of Ca^2+^ into the stimulated cells [46]. Here we verified the functionality and activation of the TRPA1 channel in human chondrocytes by measuring Ca^2+^ influx using the TRPA1 agonist AITC as well as the TRPA1 antagonist HC-030031. As shown previously, elevated intracellular Ca^2+^ concentration may affect the expression of inflammatory genes both in a direct or indirect manner [20]. In the present study, we found that TRPA1 regulated the production of inflammatory and catabolic factors, namely MMP enzymes, IL-6, and PGE_2_ in chondrocytes. IL-1-induced MMP-3, IL-6, and PGE_2_ production in the cartilage from TRPA1-deficient mice was less than half of that found in the cartilage from wild-type mice. Accordingly, the selective TRPA1 antagonist HC-030031 reduced IL-1-induced MMP-1, MMP-3, MMP-13, IL-6, and PGE_2_ production by 25–45 % in primary human OA chondrocytes. In the latter experiment, the cells were incubated in the presence of IL-1 and HC-030031 for 24 h; therefore the result may be an underestimate of the effect of total inhibition of TRPA1 in OA chondrocytes because HC-030031 is a reversible TRPA1 antagonist with a relatively short half-life [47]. These findings are supported by previous studies indicating that TRPA1 activation regulates the production of IL-1 in keratinocytes [38], IL-6 and IL-8 in synoviocytes [12], and PGE_2_ along with leukotriene B_4_ in fibroblasts and keratinocytes [48]. We have recently found that TRPA1 also regulates the expression of cyclooxygenase-2 (COX-2) [21, 27] and the production of monocyte chemotactic protein-1 (MCP-1), IL-6, IL-1β, myeloperoxidase (MPO), MIP-1α and MIP-2 in inflammatory conditions [26]. The detailed molecular mechanisms of this regulation remain, however, to be studied.

TRPA1 is shown to be involved in pain, hyperalgesia, and neurogenic inflammation [10, 16, 49, 50]. In OA-related pain, the role of TRPA1 has been investigated in studies by Moilanen et al. [27] McGaraughty et al. [51] and Okun et al. [52] using the MIA-model of OA. The two first-mentioned studies [27, 51] concluded TRPA1 to contribute to joint pain in experimental OA. In addition, Moilanen et al. [27] reported that TRPA1-deficient mice developed less severe cartilage changes following MIA injections. Accordingly, we showed here that TRPA1 is functionally expressed in chondrocytes. We also examined the possible functions of the channel by treating primary chondrocyte cultures with IL-1β and the selective antagonist HC-030031 [2, 53, 54]. Our results suggest an inflammatory and catabolic role for TRPA1 in human chondrocytes, as we found inhibition of TRPA1 to suppress the production of OA-related factors MMP-1, MMP-3, MMP-13, IL-6, and PGE_2_. These results were supported by experiments with cartilage from WT and TRPA1-deficient mice: following stimulation with IL-1β MMP-3, IL-6, and PGE_2_ production was lower in the cartilage from TRPA1-deficient mice than from WT animals. These results together suggest that TRPA1-activating factors are present in OA joints, and that TRPA1 mediates, at least partly, OA-related pain, inflammation, and cartilage destruction in neuronal and nonneuronal cells in the joint.

## Conclusions

In conclusion, we found the TRPA1 cation channel to be functionally expressed in primary human OA chondrocytes and in part to mediate inflammatory and catabolic effects, which are both original findings. The inflammatory and hypoxic environment in the OA joint is conducive to enhance the expression and activation of TRPA1. The presence and effects of TRPA1 in human OA cartilage as found in the present study, together with the previous findings on TRPA1 in experimentally induced OA [27, 51] propose an intriguing role for TRPA1 as a mediator and drug target in OA.

## Abbreviations

AITC, allyl isothiocyanate; ANOVA, analysis of variance; COX-2, cyclooxygenase-2; ELISA, enzyme-linked immunosorbent assay; H_2_O_2_, hydrogen peroxide; HIF, hypoxia-inducible factor; HRE, hypoxia response element; IL, interleukin; KO, knockout; LPS, lipopolysaccharide; MCP-1, monocyte chemotactic protein-1; MIA, monosodium iodoacetate; MIP, macrophage inflammatory protein; MMP, matrix metalloproteinase; MPO, myeloperoxidase; NF-kB, nuclear factor-kappa B; NO, nitric oxide; OA, osteoarthritis; PGE_2_, prostaglandin E_2_; qRT-PCR, quantitative reverse transcription polymerase chain reaction; SEM, standard error of the mean; TLR, toll-like receptor; TNF, tumor necrosis factor; TRPA1, transient receptor potential ankyrin 1; WT, wild-type

## Additional file

Additional file 1: Supplementary data. Supplementary information to the “Methods”. (DOCX 23 kb)

## References

[1] Chen J, Joshi SK, DiDomenico S, Perner RJ, Mikusa JP, Gauvin DM (2011). Selective blockade of TRPA1 channel attenuates pathological pain without altering noxious cold sensation or body temperature regulation. Pain..

[2] McNamara CR, Mandel-Brehm J, Bautista DM, Siemens J, Deranian KL, Zhao M (2007). TRPA1 mediates formalin-induced pain. Proc Natl Acad Sci U S A..

[3] Bautista DM, Movahed P, Hinman A, Axelsson HE, Sterner O, Hogestatt ED (2005). Pungent products from garlic activate the sensory ion channel TRPA1. Proc Natl Acad Sci U S A..

[4] Bandell M, Story GM, Hwang SW, Viswanath V, Eid SR, Petrus MJ (2004). Noxious cold ion channel TRPA1 is activated by pungent compounds and bradykinin. Neuron..

[5] Jordt SE, Bautista DM, Chuang HH, McKemy DD, Zygmunt PM, Hogestatt ED (2004). Mustard oils and cannabinoids excite sensory nerve fibres through the TRP channel ANKTM1. Nature..

[6] Jaquemar D, Schenker T, Trueb B (1999). An ankyrin-like protein with transmembrane domains is specifically lost after oncogenic transformation of human fibroblasts. J Biol Chem..

[7] Story GM, Peier AM, Reeve AJ, Eid SR, Mosbacher J, Hricik TR (2003). ANKTM1, a TRP-like channel expressed in nociceptive neurons, is activated by cold temperatures. Cell..

[8] Nilius B, Appendino G, Owsianik G (2012). The transient receptor potential channel TRPA1: from gene to pathophysiology. Pflugers Arch..

[9] Bautista DM, Jordt SE, Nikai T, Tsuruda PR, Read AJ, Poblete J (2006). TRPA1 mediates the inflammatory actions of environmental irritants and proalgesic agents. Cell..

[10] Koivisto A, Chapman H, Jalava N, Korjamo T, Saarnilehto M, Lindstedt K (2014). TRPA1: a transducer and amplifier of pain and inflammation. Basic Clin Pharmacol Toxicol..

[11] Anand U, Otto WR, Facer P, Zebda N, Selmer I, Gunthorpe MJ (2008). TRPA1 receptor localisation in the human peripheral nervous system and functional studies in cultured human and rat sensory neurons. Neurosci Lett..

[12] Hatano N, Itoh Y, Suzuki H, Muraki Y, Hayashi H, Onozaki K (2012). Hypoxia-inducible factor-1alpha (HIF1alpha) switches on transient receptor potential ankyrin repeat 1 (TRPA1) gene expression via a hypoxia response element-like motif to modulate cytokine release. J Biol Chem..

[13] Yoshida T, Inoue R, Morii T, Takahashi N, Yamamoto S, Hara Y (2006). Nitric oxide activates TRP channels by cysteine S-nitrosylation. Nat Chem Biol..

[14] Andersson DA, Gentry C, Moss S, Bevan S (2008). Transient receptor potential A1 is a sensory receptor for multiple products of oxidative stress. J Neurosci..

[15] Taylor-Clark TE, Ghatta S, Bettner W, Undem BJ (2009). Nitrooleic acid, an endogenous product of nitrative stress, activates nociceptive sensory nerves via the direct activation of TRPA1. Mol Pharmacol..

[16] Zygmunt PM, Högestätt ED (2014). Trpa1. Handb Exp Pharmacol.

[17] Wang YY, Chang RB, Waters HN, McKemy DD, Liman ER (2008). The nociceptor ion channel TRPA1 is potentiated and inactivated by permeating calcium ions. J Biol Chem..

[18] Jakobsson PJ (2010). Pain: how macrophages mediate inflammatory pain via ATP signaling. Nat Rev Rheumatol..

[19] Korhonen R, Kankaanranta H, Lahti A, Lahde M, Knowles RG, Moilanen E (2001). Bi-directional effects of the elevation of intracellular calcium on the expression of inducible nitric oxide synthase in J774 macrophages exposed to low and to high concentrations of endotoxin. Biochem J..

[20] Berridge MJ, Lipp P, Bootman MD (2000). The versatility and universality of calcium signalling. Nat Rev Mol Cell Biol..

[21] Moilanen LJ, Laavola M, Kukkonen M, Korhonen R, Leppanen T, Hogestatt ED (2012). TRPA1 contributes to the acute inflammatory response and mediates carrageenan-induced paw edema in the mouse. Sci Rep..

[22] Fernandes ES, Russell FA, Spina D, McDougall JJ, Graepel R, Gentry C (2011). A distinct role for transient receptor potential ankyrin 1, in addition to transient receptor potential vanilloid 1, in tumor necrosis factor alpha-induced inflammatory hyperalgesia and Freund's complete adjuvant-induced monarthritis. Arthritis Rheum..

[23] Caceres AI, Brackmann M, Elia MD, Bessac BF, del Camino D, D'Amours M (2009). A sensory neuronal ion channel essential for airway inflammation and hyperreactivity in asthma. Proc Natl Acad Sci U S A..

[24] Hox V, Vanoirbeek JA, Alpizar YA, Voedisch S, Callebaut I, Bobic S (2013). Crucial role of transient receptor potential ankyrin 1 and mast cells in induction of nonallergic airway hyperreactivity in mice. Am J Respir Crit Care Med..

[25] Trevisan G, Hoffmeister C, Rossato MF, Oliveira SM, Silva MA, Ineu RP (2013). Transient receptor potential ankyrin 1 receptor stimulation by hydrogen peroxide is critical to trigger pain during monosodium urate-induced inflammation in rodents. Arthritis Rheum..

[26] Moilanen LJ, Hämäläinen M, Lehtimäki L, Nieminen RM, Moilanen E (2015). Urate crystal induced inflammation and joint pain are reduced in transient receptor potential ankyrin 1 deficient mice--potential role for transient receptor potential ankyrin 1 in gout. PLoS One..

[27] Moilanen LJ, Hämäläinen M, Nummenmaa E, Ilmarinen P, Vuolteenaho K, Nieminen RM (2015). Monosodium iodoacetate-induced inflammation and joint pain are reduced in TRPA1 deficient mice – potential role of TRPA1 in osteoarthritis. Osteoarth Cartilage..

[28] Kapoor M, Martel-Pelletier J, Lajeunesse D, Pelletier JP, Fahmi H (2011). Role of proinflammatory cytokines in the pathophysiology of osteoarthritis. Nat Rev Rheumatol..

[29] Glyn-Jones S, Palmer AJ, Agricola R, Price AJ, Vincent TL, Weinans H (2015). Osteoarthritis. Lancet..

[30] Nassini R, Pedretti P, Moretto N, Fusi C, Carnini C, Facchinetti F (2012). Transient receptor potential ankyrin 1 channel localized to non-neuronal airway cells promotes non-neurogenic inflammation. PLoS One..

[31] Pfander D, Gelse K (2007). Hypoxia and osteoarthritis: how chondrocytes survive hypoxic environments. Curr Opin Rheumatol..

[32] Nummenmaa E, Hämäläinen M, Moilanen T, Vuolteenaho K, Moilanen E (2015). Effects of FGF-2 and FGF receptor antagonists on MMP enzymes, aggrecan, and type II collagen in primary human OA chondrocytes. Scand J Rheumatol..

[33] Altman R, Asch E, Bloch D, Bole G, Borenstein D, Brandt K (1986). Development of criteria for the classification and reporting of osteoarthritis. Classification of osteoarthritis of the knee. Diagnostic and Therapeutic Criteria Committee of the American Rheumatism Association. Arthritis Rheum.

[34] Goldring MB, Birkhead JR, Suen LF, Yamin R, Mizuno S, Glowacki J (1994). Interleukin-1 beta-modulated gene expression in immortalized human chondrocytes. J Clin Invest..

[35] Graham FL, Smiley J, Russell WC, Nairn R (1977). Characteristics of a human cell line transformed by DNA from human adenovirus type 5. J Gen Virol..

[36] Moilanen LJ, Hämäläinen M, Lehtimäki L, Nieminen RM, Muraki K, Moilanen E (2016). Pinosylvin inhibits TRPA1-induced calcium influx in vitro and TRPA1-mediated acute paw inflammation in vivo. Basic Clin Pharmacol Toxicol.

[37] Tsutsumi M, Denda S, Ikeyama K, Goto M, Denda M (2010). Exposure to low temperature induces elevation of intracellular calcium in cultured human keratinocytes. J Invest Dermatol..

[38] Atoyan R, Shander D, Botchkareva NV (2009). Non-neuronal expression of transient receptor potential type A1 (TRPA1) in human skin. J Invest Dermatol..

[39] Kochukov MY, McNearney TA, Fu Y, Westlund KN (2006). Thermosensitive TRP ion channels mediate cytosolic calcium response in human synoviocytes. Am J Physiol Cell Physiol..

[40] Fernandes ES, Fernandes MA, Keeble JE (2012). The functions of TRPA1 and TRPV1: moving away from sensory nerves. Br J Pharmacol..

[41] Ellman MB, Yan D, Ahmadinia K, Chen D, An HS, Im HJ (2013). Fibroblast growth factor control of cartilage homeostasis. J Cell Biochem..

[42] Hellwig-Burgel T, Rutkowski K, Metzen E, Fandrey J, Jelkmann W (1999). Interleukin-1beta and tumor necrosis factor-alpha stimulate DNA binding of hypoxia-inducible factor-1. Blood..

[43] Rius J, Guma M, Schachtrup C, Akassoglou K, Zinkernagel AS, Nizet V (2008). NF-kappaB links innate immunity to the hypoxic response through transcriptional regulation of HIF-1alpha. Nature..

[44] Koskinen A, Vuolteenaho K, Moilanen T, Moilanen E (2014). Resistin as a factor in osteoarthritis: synovial fluid resistin concentrations correlate positively with interleukin 6 and matrix metalloproteinases MMP-1 and MMP-3. Scand J Rheumatol..

[45] Tarkowski A, Bjersing J, Shestakov A, Bokarewa MI (2010). Resistin competes with lipopolysaccharide for binding to toll-like receptor 4. J Cell Mol Med..

[46] Nilius B (2007). Transient receptor potential (TRP) cation channels: rewarding unique proteins. Bull Mem Acad R Med Belg..

[47] Rech JC, Eckert WA, Maher MP, Banke T, Bhattacharya A, Wickenden AD (2010). Recent advances in the biology and medicinal chemistry of TRPA1. Future Med Chem..

[48] Jain A, Bronneke S, Kolbe L, Stab F, Wenck H, Neufang G (2011). TRP-channel-specific cutaneous eicosanoid release patterns. Pain..

[49] Baraldi PG, Preti D, Materazzi S, Geppetti P (2010). Transient receptor potential ankyrin 1 (TRPA1) channel as emerging target for novel analgesics and anti-inflammatory agents. J Med Chem..

[50] Wei H, Koivisto A, Pertovaara A (2010). Spinal TRPA1 ion channels contribute to cutaneous neurogenic inflammation in the rat. Neurosci Lett..

[51] McGaraughty S, Chu KL, Perner RJ, Didomenico S, Kort ME, Kym PR (2010). TRPA1 modulation of spontaneous and mechanically evoked firing of spinal neurons in uninjured, osteoarthritic, and inflamed rats. Mol Pain..

[52] Okun A, Liu P, Davis P, Ren J, Remeniuk B, Brion T (2012). Afferent drive elicits ongoing pain in a model of advanced osteoarthritis. Pain..

[53] Eid SR, Crown ED, Moore EL, Liang HA, Choong KC, Dima S (2008). HC-030031, a TRPA1 selective antagonist, attenuates inflammatory- and neuropathy-induced mechanical hypersensitivity. Mol Pain.

[54] Taylor-Clark TE, Undem BJ, Macglashan DW, Ghatta S, Carr MJ, McAlexander MA (2008). Prostaglandin-induced activation of nociceptive neurons via direct interaction with transient receptor potential A1 (TRPA1). Mol Pharmacol..

