# Supplementary material for: Transient receptor potential ankyrin 1 (TRPA1) is functionally expressed in primary human osteoarthritic chondrocytes
Source: Arthritis Res Ther. 2016 Aug 11;18:185. doi: 10.1186/s13075-016-1080-4 (PMC4982008; doi:10.1186/s13075-016-1080-4)
Supplement: Additional file 1: — Supplementary data. Supplementary information to the “Methods”. (DOCX 23 kb) [file 13075_2016_1080_MOESM1_ESM.docx]

**SUPPLEMENTARY DATA**

**Supplementary Information to the Methods**

**Cell culture**

Primary human OA chondrocytes were isolated as descried previously [1]. Leftover pieces of OA cartilage from knee joint replacement surgery were used. The patients in this study fulfilled the American College of Rheumatology classification criteria for OA [2]. The study was approved by the Ethics Committee of Tampere University Hospital, Tampere, Finland, and carried out in accordance with the Declaration of Helsinki. Written informed consent was obtained from the patients. Full-thickness pieces of articular cartilage from femoral condyles, tibial plateaus and patellar surfaces showing macroscopic features of early OA were removed aseptically from subchondral bone with a scalpel, cut into small pieces and washed with PBS. Chondrocytes were then isolated by enzymatic digestion for 16 h at 37 °C in a shaker by using a collagenase enzyme blend (0.25 mg/ml Liberase TM Research Grade medium; Roche, Mannheim, Germany). Isolated chondrocytes were washed and plated on 24-well plates for mRNA measurements and immunoassays, on 6-well plates for Western Blot, and on 96-well plates for Ca^2+^ assays in culture medium [DMEM (Sigma-Aldrich, St. Louis, MO, USA) supplemented with penicillin (100 U/ml), streptomycin (100 μg/ml) and amphotericin B (250 ng/ml) all from Gibco/Life Technologies, Carlsbad, CA, USA) containing 10 % fetal bovine serum (Lonza, Verviers, Belgium)]. During experiments the cells were treated with IL-1β (R&D Systems Europe Ltd, Abingdon, UK), IL-17 (R&D Systems Europe Ltd), resistin (BioVision Inc., Milpitas, CA, USA), LPS (Sigma-Aldrich), the TRPA1 antagonist HC-030031 (Sigma-Aldrich) or with combinations of these compounds as indicated.

Immortalized human T/C28a2 chondrocytes [3] were cultured in DMEM (Sigma-Aldrich,) and Ham’s F-12 medium (Lonza) (1:1, v/v) containing 10 % heat-inactivated fetal bovine serum (Lonza), 100 U/mL penicillin, 100 μg/mL streptomycin, and 250 ng/mL amphotericin B (all from Gibco/Life Technologies). Cells were grown on 24-well plates for mRNA measurements, on 6-well plates for Western Blot measurements, and on 96-well plates for Ca^2+^ assays. During experiments the cells were treated with IL-1β (R&D Systems Europe Ltd), IL-17 (R&D Systems Europe Ltd), LPS (Sigma-Aldrich), HC-030031 (Sigma-Aldrich) or with combinations of these compounds as indicated.

HEK 293 human embryonic kidney cells (American Type Culture Collection, Manassas, VA, USA) were cultured in Eagle’s Minimum Essential Medium (EMEM) supplemented with 10 % heat-inactivated fetal bovine serum, sodium bicarbonate (1.5%), sodium pyruvate (1 mM), non-essential amino acids (1 mM each) (all from Lonza), penicillin (100 U/ml), streptomycin (100 mg/ml) and amphotericin B (all from Gibco/Life Technologies) at 37 °C in 5 % CO_2_. The cells were transfected using 0.42 mg/cm^2^ of human TRPA1 plasmid DNA (pCMV6-XL4 from Origene Rockville, MD, USA) with lipofectamine 2000 (Invitrogen/Life Technologies, Carlsbad, CA, USA) according to the manufacturer’s directions. Cells were grown on 6-well plates for Western Blot analysis, and on 96-well plates for Ca^2+^ assays.

**Mouse Cartilage Culture**

After mice were euthanized, full-thickness articular cartilage from the femoral heads was removed, and incubated at 37°C in 5% CO_2_ in DMEM supplemented with penicillin (100 U/ml), streptomycin (100 μg/ml), and amphotericin B (250 ng/ml, all from Gibco/Life Technologies) containing 10 % fetal bovine serum. The cartilage pieces were exposed to IL-1β (100 pg/ml, R&D Systems Europe Ltd.) for 42 h and thereafter culture media were collected and MMP-3, IL-6 and PGE_2_ concentrations were measured by immunoassay.

**Western Blot Measurements**

After the cell culture experiments, cells were rapidly washed with ice-cold PBS and solubilized in cold lysis buffer containing Tris-HCl (50 mM, pH 8), NaCl (150 mM), EDTA (5 mM), Nonidet P-40 (1%), Na deoxylate (0.5%), SDS (0.1%), phenylmethylsulfonyl fluoride (0.5 mM), sodiumorthovanadate (1 mM), leupeptin (20 µg/ml), aprotinin (50 µg/ml), NaF (5 mM) and sodiumpyrophosphate (2 mM). The cells were incubated in the lysis buffer at 4 °C for 30 min, vortexing every 5 min, and then centrifuged (12 000 g, 4 °C, 15 min) and supernatants were collected and stored at -70 °C. An aliquot of the supernatant was used to determine protein concentration by the Coomassie blue method [4]. Prior to Western Blot TRPA1 proteins were immunoprecipitated with TRPA1 antibody NB110-40763 (2 µg, NovusBiologicals, LCC, Littleton, CO, USA) using protein A/G PLUS-Agarose (sc-2003, Santa Cruz Biotechnology, Inc., Santa Cruz, CA, USA), according to the manufacturer’s instructions. The immunoprecipitated TRPA1 protein samples were loaded onto an 8 % SDS-polyacrylamide electrophoresis gel and electrophoresed for 5 h at 100 V in a buffer containing Tris–HCl (95 mM), glycine (960 mM), and SDS (0.5%). After electrophoresis, the proteins were transferred to a nitrocellulose membrane (Novex, Life Technologies). After transfer, the membrane was blocked in TBS/T (20 mM Tris–base pH 7.6, 150 mM NaCl, 0.1% Tween-20) containing 5 % nonfat milk for 1 h at room temperature and incubated in the blocking solution at 4 °C overnight with the primary antibody for TRPA1 (NB110-40763, 1:1000, NovusBiologicals). On the next day, the membrane was incubated in the blocking solution for 1 h at room temperature with the respective secondary antibody for TRPA1 (goat anti-rabbit HRP-conjugate sc-2004, 1:10 000, Santa Cruz Biotechnology, Inc.). Bound antibody was detected using Super Signal® West Dura chemiluminescent substrate (Pierce, Rockford, IL, USA) and Image Quant LAS 4000 mini imaging system (GE Healthcare Bio-Sciences AB, Uppsala, Sweden).

**RNA extraction and quantitative RT-PCR**

RNA extraction and quantitative RT-PCR (qRT-PCR) was carried out as previously described [1]. At the indicated time points, culture medium was collected and total RNA of the cell monolayers was extracted with GenElute Mammalian Total RNA Miniprep kit (Sigma-Aldrich). The amount of RNA was measured with a spectrophotometer. Total RNA was then reverse-transcribed to cDNA using Maxima First Strand cDNA synthesis kit (Fermentas UAB, Vilnius, Lithuania) in 10 µl reaction volume. After the transcription reaction, the obtained cDNA was diluted 1:5 with RNase-free water. Quantitative PCR was performed using TaqMan Universal PCR Master Mix and ABI Prism 7000 sequence detection system (Applied Biosystems, Foster City, CA, USA). The primer and probe sequences and concentrations for hGAPDH were optimized according to the manufacturer’s guidelines in TaqMan® Universal PCR Master Mix Protocol part number 4304449 revision C (Applied Biosystems) and were: 5′-AAGGTCGGAGTCAACGGATTT-3’ (human GAPDH, forward, 300 nM), 5’-GCAACAATATCCACTTTACCAGAGTTAA-3’ (human GAPDH, reverse, 300 nM), and 5’-CGCCTGGTCACCAGGGCTGC-3’ (human GAPDH, probe, 150 nM, containing 6-FAM as 5′-reporter dye and TAMRA as 3′-quencher) (obtained from Metabion, Martinsried, Germany). TaqMan Gene Expression assay for human TRPA1 (Hs00175798_m1) was purchased from Life Technologies (Life Technologies Europe BV, Bleiswijk, the Netherlands). PCR cycling parameters were: incubation at 50 °C for 2 min, incubation at 95 °C for 10 min, and thereafter 40 cycles of denaturation at 95 °C for 15 s and annealing and extension at 60 °C for 1 min. The relative mRNA levels were quantified using a standard curve method as described in the Applied Biosystems User Bulletin. For the TaqMan Gene Expression assay the ^ΔΔ^Ct method was used. When calculating results, mRNA expression levels were first normalized against GAPDH mRNA levels.

**References**

1. Nummenmaa E, Hämäläinen M, Moilanen T, Vuolteenaho K, Moilanen E. Effects of FGF-2 and FGF receptor antagonists on MMP enzymes, aggrecan, and type II collagen in primary human OA chondrocytes. Scand J Rheumatol 2015;44:321-330.

2. Altman R, Asch E, Bloch D, Bole G, Borenstein D, Brandt K, et al. Development of criteria for the classification and reporting of osteoarthritis. Classification of osteoarthritis of the knee. Diagnostic and Therapeutic Criteria Committee of the American Rheumatism Association. Arthritis Rheum 1986;29:1039-1049.

3. Goldring MB, Birkhead JR, Suen LF, Yamin R, Mizuno S, Glowacki J, et al. Interleukin-1 beta-modulated gene expression in immortalized human chondrocytes. J Clin Invest 1994;94:2307-2316.

4. Bradford MM. A rapid and sensitive method for the quantitation of microgram quantities of protein utilizing the principle of protein-dye binding. Anal Biochem 1976;72:248-254.
